# Reduced Expression of SATB2 in Colorectal Cancer and Its Association with Demographic and Clinicopathological Parameters

**DOI:** 10.3390/ijms26052374

**Published:** 2025-03-06

**Authors:** Anna Ewa Kowalczyk, Agnieszka Śliwińska-Jewsiewicka, Bartłomiej Emil Kraziński, Aleksandra Piotrowska, Jędrzej Grzegrzółka, Janusz Godlewski, Piotr Dzięgiel, Zbigniew Kmieć

**Affiliations:** 1 Department of Anatomy and Histology, School of Medicine, Collegium Medicum, University of Warmia and Mazury in Olsztyn, 10-082 Olsztyn, Poland; a.sliwinska@uwm.edu.pl (A.Ś.-J.); bartlomiej.krazinski@uwm.edu.pl (B.E.K.); janusz.godlewski@uwm.edu.pl (J.G.); zbigniew.kmiec@uwm.edu.pl (Z.K.); 2 Division of Histology and Embryology, Department of Human Morphology and Embryology, Wroclaw Medical University, 50-368 Wroclaw, Poland; aleksandra.piotrowska@umw.edu.pl (A.P.); jedrzej.grzegrzolka@umw.edu.pl (J.G.); piotr.dziegiel@umw.edu.pl (P.D.)

**Keywords:** SATB2, colorectal cancer, qPCR, immunohistochemistry, demographic and clinicopathological parameters, survival

## Abstract

Special AT-rich sequence-binding protein 2 (SATB2), as a nuclear matrix-associated protein and transcription factor engaged in chromatin remodeling and the regulation of gene expression, plays an important role in growth and development processes. SATB2 has been shown to have tissue-specific expression, also related to some cancers, including colorectal cancer (CRC). The aim of this study was to compare *SATB2* gene expression in tumor and matched non-involved colorectal tissues obtained from CRC patients, and to investigate its association with clinicopathological and demographic parameters, as well as patients’ overall survival. *SATB2* mRNA levels in the tested tissues were assessed by quantitative polymerase chain reaction, while SATB2 protein expression was determined by immunohistochemistry. We found that the average levels of both *SATB2* mRNA and protein were significantly lower in tumor specimens than in matched non-involved colon tissues. Moreover, SATB2 immunoreactivity was associated with patients’ sex, tumor localization, and grade of differentiation. Lower immunoreactivity of SATB2 protein was noted in high-grade tumors, in women, and in tumors located in the cecum, ascending, and transverse colon. However, the results of the present study did not show an association between *SATB2* expression levels and patients’ overall survival. Our findings indicate the involvement of impaired SATB2 expression, significantly reduced in high-grading tumors, in the pathogenesis of CRC, while its sex- and localization-specificity should be further elucidated.

## 1. Introduction

Colorectal cancer (CRC) ranks second in terms of mortality and third in terms of incidence worldwide, accounting for nearly one in ten cancer cases and deaths [1]. CRCs represent a highly heterogeneous group of diseases caused by a wide range of mutagenic agents. CRC usually develops from glandular epithelial cells of the large intestine when they acquire a series of epigenetic or genetic changes that confer on them abnormal characteristics, such as increased proliferation and survival [2,3]. Various molecular pathways influence individual susceptibility to cancer and determine responsiveness or resistance to anticancer agents [3]. Studies on the molecular basis of CRC may contribute to the identification of molecular markers, enabling early detection as well as more efficient treatment of CRC.

Special AT-rich sequence-binding protein (SATB2), encoded by the *SATB2* gene located on 2q33, is a highly evolutionary conserved protein composed of 733 amino acids with a molecular weight of 82.5 kDa [4,5]. SATB2 binds to specific nuclear matrix attachment regions and participates in chromatin remodeling and transcriptional regulation. This transcription factor and epigenetic regulator influences gene expression and is involved in growth and developmental processes. SATB2 plays important roles in the craniofacial and skeleton development, osteoblast differentiation, modulation of immunoglobulin gene expression, and neurogenesis [4,6]. Moreover, the results of studies conducted in the last decade have indicated that SATB2 is involved in processes related to carcinogenesis [4,5]. Nevertheless, it has also been noted that SATB2 functions may be related to the type of cancer. Also, the expression profile of SATB2 in tumor tissues compared to normal tissues varies by tumor type. In cancers such as osteosarcoma, hepatocellular carcinoma (HCC), head and neck squamous cell carcinoma (HNSCC), or breast cancer, SATB2 expression levels have been observed to be higher than in normal tissues and associated with a poor prognosis. The effects of SATB2 action on these cancers’ cell lines promoted their growth and progression [4,7,8,9,10,11,12,13]. On the contrary, SATB2 may exert anti-cancerous effects since its reduced expression associated with a worse prognosis has been reported in laryngeal, esophageal, non-small-cell lung, ovarian endometriod, and clear cell renal cell cancers [4,14,15,16,17,18]. Furthermore, higher levels of *SATB2* were found in HCC cells derived from African Americans than in those from Caucasian Americans and were associated with higher growth rate, colony formation, cell viability, and epithelial-mesenchymal transition (EMT) characteristics [9]. So, it seems that SATB2 expression is not only tissue- and cancer-type dependent but may also be racial/ethnic-specific.

Despite numerous studies conducted, the level of SATB2 expression in CRC tissues and the involvement of SATB2 in the pathogenesis of CRC remain controversial. Some authors found increased SATB2 expression in CRC and presented this protein as an oncogenic factor associated with a worse prognosis [19,20,21], while others reported decreased levels of SATB2 in CRC that was associated with poor prognosis [22,23,24,25,26,27,28]. Similarly, there are discrepant data on the correlations between SATB2 levels in CRC and demographic and clinicopathological parameters [22,23,24,29,30,31,32,33]. Thus, the aim of our research was to compare the levels of *SATB2* mRNA and protein in CRC and matched non-involved colon specimens of Polish patients and examine their association with clinicopathological parameters, as well as with the overall survival (OS) of patients, in order to assess the prognostic value.

## 2. Results

### 2.1. Reduced SATB2 mRNA Levels in CRC Tissues

**Figure 1:**
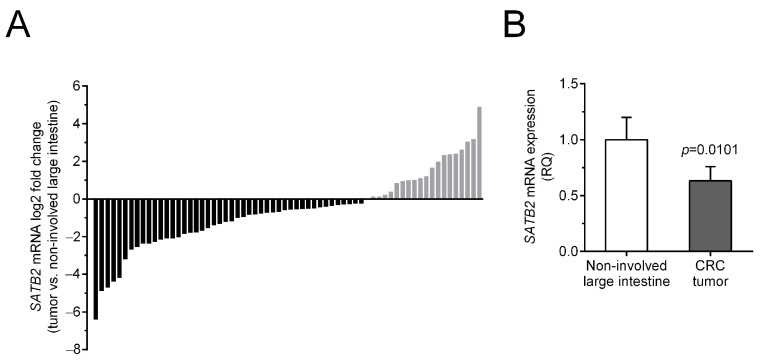
*SATB2* mRNA expression in the tumor and non-involved colon tissues from patients with colorectal cancer (CRC) assessed by qPCR. (**A**) mRNA levels of *SATB2* in individual patients’ tumors are demonstrated in relation to the *SATB2* mRNA expression in corresponding non-involved colon specimens. (**B**) The average content of *SATB2* mRNA (mean ± SEM) in CRC tumors versus matched non-involved colorectal tissues (1.0), *n* = 66.

To compare *SATB2* mRNA levels in tumor and non-involved colorectal tissues, matched tissue samples from 66 CRC patients were analyzed by quantitative polymerase chain reaction (qPCR). *SATB2* transcripts were found in all tissue specimens studied. Forty-six (69.7%) CRC tissue specimens showed decreased relative *SATB2* mRNA levels (tumor vs. matched non-involved colorectal mucosa), while 20 (30.3%) showed increased levels of *SATB2* transcripts (Table 1, Figure 1A).

The average level of *SATB2* mRNA expression was significantly reduced in the CRC samples compared to non-involved specimens (0.63 ± 0.13 vs. 1.00 ± 0.20, respectively; *p* = 0.0101; Figure 1B). Analysis of the results obtained by qPCR showed no association of *SATB2* mRNA expression levels with demographic and clinicopathological parameters (age of patients at diagnosis, sex, tumor localization, grade, depth of invasion, lymph node involvement, presence of metastases, TNM disease stage; *p* > 0.05; Table 1).

### 2.2. Downregulated SATB2 Immunoreactivity (SATB2-Ir) in CRC Tissues and Its Association with Sex, Tumor Localization, and Histological Grade

**Figure 2:**
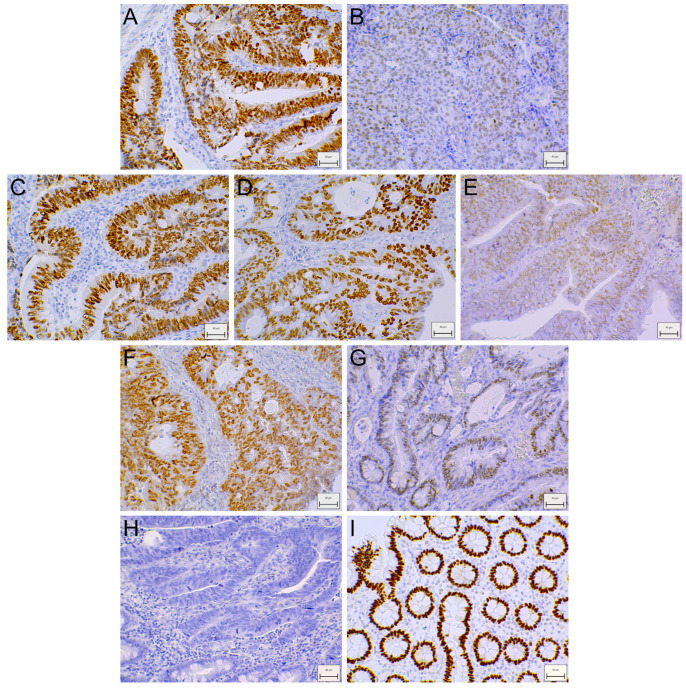
SATB2 immunoreactivity in representative colorectal cancer (CRC) (**A**–**G**) and non-involved (**I**) tissues. Heterogeneous nuclear SATB2 immunostaining depending on differentiation grade: G2 (**A**) and G3 (**B**), tumor localization: rectum (**C**), descending and sigmoid colon (**D**) and cecum, ascending and transverse colon (**E**), sex: men (**F**) and women (**G**). Negative staining in CRC (**H**). Magnification ×200. Scale bar: 40 µm.

Immunohistochemical (IHC) study of the SATB2 protein localization and level included sections of tumor and matched non-involved colon tissues of 104 CRC patients. SATB2-Ir was observed in the nuclei of epithelial cells (Figure 2I), as well as cancer cells (Figure 2A–G). Moreover, SATB2-Ir was present in some stromal cells (not included in the analyses).

Nuclear SATB2 immunostaining was observed in epithelial cells in 98/104 (94.2%) of non-involved colorectal tissues, while in cancer cells, SATB2-Ir was detected in 84/104 (80.8%) CRC cases. Assuming that the immunoreactivity score > 2.0 defined tumors with high SATB2-Ir, we found that IHC staining of SATB2 was absent or low in 30/104 (28.8%) and high in 74/104 (71.2%) CRC specimens (Table 2). Among 104 matched non-involved colorectal samples tested, SATB2-Ir was low in 11 (10.6%) and high in 93 (89.4%) cases.

**Figure 3:**
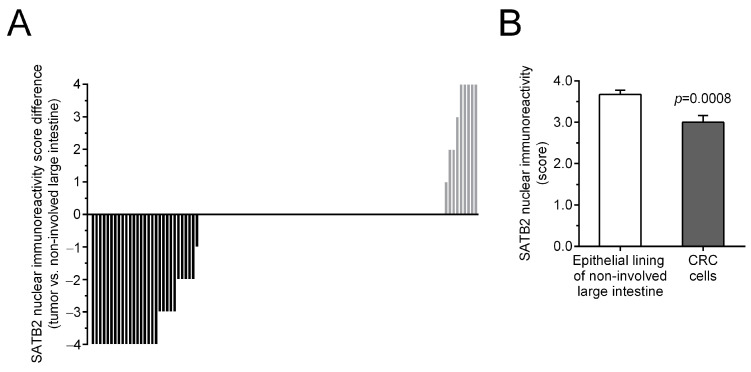
Nuclear SATB2 immunoreactivity (SATB2-Ir) in non-involved and tumor tissues from patients with colorectal cancer (CRC), *n* = 104. (**A**) SATB2-Ir in cancer cells of individual patients is presented in relation to the level of SATB2-Ir in epithelial cells of matched non-involved colon mucosa. (**B**) The average nuclear SATB2-Ir (mean ± SEM) in colonocytes and CRC cells.

The level of nuclear SATB2 immunostaining in tumors of individual CRC patients in relation to the level of SATB2 immunostaining in matched non-involved colorectal tissues is shown in Figure 3A. The average score of SATB2-Ir was lower in CRC cells than in the epithelial cells of non-involved mucosa (3.00 ± 0.16 vs. 3.67 ± 0.10, respectively; *p* = 0.0008; Figure 3B).

**Figure 4:**
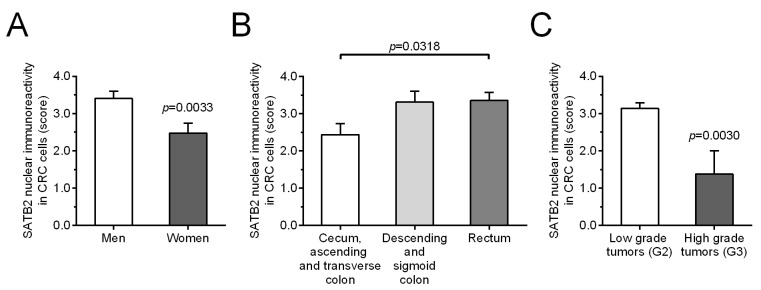
The average nuclear SATB2 immunoreactivity in colorectal cancer specimens in relation to the patients’ sex (**A**), tumor localization (**B**), and differentiation grade (**C**). Bars represent mean ± SEM.

We also examined relationships between SATB2 immunostaining levels and selected clinicopathological and demographic parameters. SATB2-Ir was lower in CRC samples derived from: (i) women [*p* = 0.001, the Fisher’s exact test, Table 2; confirmed by the unpaired *t* test: 2.48 ± 0.26 vs. 3.41 ± 0.19 (women vs. men), *p* = 0.0033; Figure 4A], (ii) cecum, ascending and transverse colon [*p* = 0.0365, the Fisher’s exact test, Table 2; confirmed by the one-way ANOVA followed by Tukey’s multiple comparison test: 2.44 ± 0.30 vs. 3.36 ± 0.22 (vs. rectum), *p* = 0.0318, and vs. 3.31 ± 0.29 (vs. descending and sigmoid colon), *p* = 0.0827; Figure 4B], (iii) patients with high grade tumors [*p* = 0.0068, the Fisher’s exact test, Table 2; confirmed by the unpaired *t* test: 1.38 ± 0.63 vs. 3.14 ± 0.16 (high vs. low grade), *p* = 0.003; Figure 4C]. No significant association was found between SATB2-Ir and age of patients at diagnosis, depth of invasion, lymph node involvement, presence of metastases and TNM disease stage (*p* > 0.05; Table 2).

### 2.3. No Association of SATB2 mRNA and Protein Levels with Overall Survival of CRC Patients

**Figure 5:**
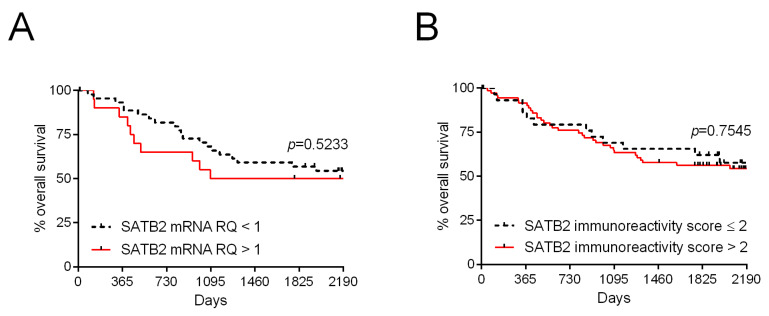
Kaplan–Meier survival curves of CRC patients with regard to *SATB2* mRNA levels (**A**) and SATB2 immunoreactivity (**B**).

In order to evaluate the prognostic significance of *SATB2* gene expression, patients were followed-up for 61.1 months. During this period, 44 (42.3%) patients died. The levels of *SATB2* mRNA and SATB2 immunostaining were not related to OS of the patients (Figure 5A and Figure 5B, respectively; Table 3).

Univariate Cox proportional hazards regression revealed that the advanced age at diagnosis (*p* = 0.0082, Table 3), depth of invasion (T3 + T4, *p* = 0.0308; Table 3), lymph node involvement (*p* = 0.0001; Table 3), presence of distant metastases (*p* < 0.0001; Table 3), and TNM disease stage (III–IV; *p* < 0.0001; Table 3) were associated with poor survival rates of the patients. Subsequent multivariate analysis confirmed that patient age (*p* = 0.014; Table 3) and the presence of distant metastases (*p* = 0.0005; Table 3) were independent prognostic factors in CRC.

## 3. Discussion

CRC is one of the most common and deadly malignancies worldwide [1]. Unfortunately, there is a progressive increase in the incidence of this cancer, especially in people younger than 50 years [1]. It has been estimated that the incidence of CRC will increase by 60% to more than 2.2 million new cases and 1.1 million cancer deaths by 2030 [34]. Therefore, there is an urgent need to discover potential biomarkers and therapeutic targets to improve diagnosis, prognosis, and to develop more effective treatments of CRC.

Genes encoding proteins associated with cancer pathogenesis often show altered expression in tumor tissues. This fact is used to search for factors involved in cancer development and progression, and to analyze their potential use as biomarkers and prognostic factors. *SATB2* is a gene whose expression has been extensively studied over the last decade. Its protein product is expressed in embryonic cells, while its expression is often low or absent in normal human tissues [4,5,35,36]. SATB2 was identified as a highly tissue type-specific protein, and its expression is found in epithelial cells of the ileum, neurons of cerebral cortex, osteoblasts, and at lower levels in subsets of lymphocytes, spermatocytes, oocytes, as well as some epithelial cells of kidney distal tubuli and collecting ducts [5,36]. Strong expression of SATB2 was detected in glandular cells of the colon, rectum, and appendix [5,36,37].

SATB2 has also been linked to cancer. Previous reports on the involvement of SATB2 in various cancers indicated a cancer-specific role and expression of SATB2 [38], identifying either its tumor-promoting function and increased expression in some cancer types [4,7,8,9,10,11,12,13] or its tumor-suppressor role and decreased expression in others [4,14,15,16,17,18]. Elevated expression of SATB2 has been shown to promote EMT in HCC cells [9], induce the transformation of normal human breast mammary epithelial cells into progenitor-like cells resulting in a malignant phenotype [12], and to enhance chemoresistance of HNSCC [13] and migration and invasion in osteosarcoma [7,8]. However, downregulated expression of SATB2 was revealed to induce EMT in non-small-cell lung carcinoma [16], and to promote cell proliferation and tumor progression ability in laryngeal carcinoma [14].

SATB2 expression was found in the vast majority of both primary and metastatic CRCs, suggesting that SATB2 may be used as a diagnostic marker to differentiate CRC from other cancer types [37,39], especially with concomitant immunostaining of cytokeratin 20 [37] or cadherin 17 [40]. We observed positive SATB2 immunostaining in 80.8% of CRC cases, which is consistent with results presented in the recent meta-analysis by Maguire et al., who reported SATB2 expression in 81% of CRCs [41]. In other studies, the range of SATB2 positivity in CRCs was 71–97% [5,29,30,31,33,37,39,40,42,43,44,45,46,47]. Some discrepancies between reports may be caused by differences in the methods (antibody clones, staining protocols) and the characteristics of the patients’ cohorts. It has been demonstrated that microsatellite instability (MSI) [5,32,33], DNA mismatch repair protein deficiency [30,31], and the presence of *BRAF* mutation [5,30,31] are related to the loss or a decrease in SATB2 expression. Moreover, the absence or presence of SATB2 expression may also depend on the CRC subtype. Low or absent SATB2 expression has been documented in the mucinous, micropapillary, medullary, and signet-ring CRC subtypes, and also in colorectal mixed adenoneuroendocrine carcinomas/neuroendocrine carcinomas (MANECs/NECs) [32,40]. When considering the reasons for discrepancies in SATB2 expression, the possible influence of race/ethnicity of patients included in the study cohort should also be taken into account, as Yu et al. found higher levels of SATB2 in HCC cells derived from African Americans than in those derived from Caucasian Americans [9].

Despite consensus on the highly specific expression of SATB2 in CRC, in contrast to other types of neoplasms, only a few authors tried to semi-quantitatively, i.e., by applying a scoring system, evaluate the intensity and range of immunoreactivity in the CRC samples in regard to patients’ clinicopathological characteristics. However, reports on SATB2 expression in CRC and its associations with clinicopathological parameters and patient survival have provided inconclusive results [20,21,22,23,24,25,26,27]. Our findings of reduced expression of SATB2 protein in CRC tissues are consistent with previous reports [22,24]. Wang et al. were the first to report decreased SATB2 expression in a group of 146 CRC patients [22]. They found high SATB2-Ir in 45% of CRC tissues, whereas the remaining 55% presented low immunoreactivity. Similarly, Gu et al. showed reduced SATB2 protein expression in tumor specimens compared to paired normal tissues [24]. Furthermore, they observed low SATB2 expression in 81% of CRC cases, while high expression was shown in 19% of cases. Interestingly, studies in European populations with a different genetic background, including this report, revealed a much lower fraction of patients with low SATB2-Ir in CRC specimens [23,32]. For example, Eberhardt et al., using the tissue microarray (TMA), found moderate and high SATB2-Ir in the majority of 527 analyzed incident colon and rectal cancers, while 28.8% of patients presented negative SATB2 staining [23]. More recently, Schmitt et al. also used the TMA to assess SATB2-Ir in a large cohort of CRC patients [32]. They found loss or low SATB2-Ir in 22.2% of CRC [32]. The results of our study indicate that the absence of or low SATB2 immunostaining in 28.8% of CRC tissue are consistent with these aforementioned reports.

In addition to reduced SATB2 protein immunoreactivity, we also observed decreased *SATB2* transcript content in tumor tissues, which is in line with the findings of other authors [22,24] and advocates a tumor suppressor role for SATB2. SATB2 has been shown to repress the divisions of CRC cells in vitro and in vivo, and to inhibit their migration and invasion via the inactivation of extracellular signal-regulated kinase 5 [25]. Moreover, SATB2 can suppress the metastasis by weakening the EMT in an in vivo model of CRC [24] and negatively regulating the stemness of CRC cells [26]. In contrast to our results and the aforementioned studies, other researchers demonstrated elevated expression of *SATB2* mRNA and protein in CRC compared to adjacent non-involved specimens, and suggested the role of *SATB2* as an oncogene promoting cell proliferation and the invasion of CRC cells [20,21]. Moreover, Yu et al. found high levels of SATB2 mRNA and protein in CRC cell lines; however, in contrast to the majority of studies, they did not detect SATB2 staining in normal colon epithelial cells [19]. Furthermore, these authors showed that SATB2 induced malignant transformation of the latter cells by generating CRC stem cell-like cells [19].

It is difficult to clearly identify the reason for the discrepancies between the results of studies on SATB2 expression in CRC. To explain the differences in findings, it would be necessary to take into account the complexity of *SATB2* expression regulation. *SATB2* expression and posttranslational modification and functions are regulated by numerous growth factors, cytokines, and non-coding RNA molecules [4]. Of the latter, attention has been given to long non-coding RNA antisense transcript of SATB2 (*lncRNA SATB2-AS1*) that can activate *SATB2* transcription and suppress CRC progression and aggressiveness. *LncRNA SATB2-AS1* was found to inhibit SATB2-dependent *Snail* transcription and EMT, however, its expression is downregulated in CRC [38]. Among microRNAs (miRs), miR-449a [20], miR-3666 [21], miR-31 [48], miR-34c-5p [24], and miR-182 [49] have been indicated to repress *SATB2* expression in CRC cell lines. miR-3666 and miR-449a are downregulated [20,21], while miR-31 and miR-182 are upregulated [48,49] in CRC. Thus, the differences in SATB2 expression levels in CRC may be due to the impaired expression of its regulators. Moreover, carcinogenic metals (nickel, arsenic, vanadium, and chromium) upregulated *SATB2* gene expression in transformed human lung cells [50], indicating the involvement of SATB2 induction in metal-induced carcinogenesis.

In all likelihood, we were the first to analyze the associations of *SATB2* mRNA levels in CRC with clinicopathological features. However, our study showed no statistically significant association between *SATB2* mRNA expression and the clinicopathological parameters studied. In agreement with some previous reports [23,29,30,31,32,33,43,51], we noted an association between SATB2 protein expression in CRC tissues and the grade of histological differentiation, observing reduced SATB2-Ir in high-grade tumors. However, in contrast to our and the aforementioned findings, Wang et al. [22] and Gu et al. [24] showed no relationship between tumor differentiation and SATB2 protein expression in Chinese patients with CRC. One of the reasons for this discrepancy could be the different genetic background of the patients [22,24]. Our finding of reduced SATB2 expression in right-sided tumors (located in the cecum, ascending and transverse colon) is consistent with some previous reports [5,29,31,32] and contradictory to several others in which no such relationship was observed [22,23,24,30,33,47]. This can be explained by the molecular characteristics of right-sided colon cancers, which are different from those of left-sided colon cancers and rectal cancers [52]. Right-sided tumors are more likely to be MSI-high and have a *BRAF* mutation [52] and, as mentioned earlier, these are factors associated with absent or reduced SATB2 expression [30,31,32]. Tumors with right-sided location are more common in women [52]. Surprisingly, we also revealed a previously undemonstrated association of SATB2 expression with sex, showing lower SATB2 protein immunoreactivity in CRC tumors from women. Our study showed no association of SATB2 expression with other parameters such as age, depth of invasion, lymph node metastasis, distant metastases, or TNM stage, which corresponds to the results of Liu et al. [47] and partially of Mezheyeuski et al. [29]. Contrary to our findings, some researchers have shown an association of SATB2 expression with depth of invasion [23], lymph node metastasis [22,23,24,30,33,51], distant metastases [22,24,30,51], and TNM stage [23,30,33,51]. The lack of concordance between the results of our analysis of associations between clinicopathological data and *SATB2* mRNA and protein levels may result from post-transcriptional and post-translational modifications, as well as the difference in the number of specimens studied by the qPCR and IHC methods.

It was shown in the majority of studies that reduced SATB2 expression in CRC was associated with poor overall survival [5,22,23,24,29,30,33], with the one exception in which it was associated with good prognosis [20]. Our results did not show that SATB2 is an independent prognostic factor, as we did not observe a significant association between *SATB2* gene expression (neither at mRNA nor protein level) and patient survival, which is consistent with another report [47]. The reason for the discrepancy in the results of the different groups may lie in the molecular characteristics of the tumors included in the study. SATB2 expression has been demonstrated to have no effect on patient survival in MSI, mismatch repair protein proficient, *BRAF*-mutated, or *KRAS*-mutated tumors [23,31]. Another factor that may contribute to the observed differences in the prognostic potential of SATB2 is the location of the tumors studied. Eberhard et al. found a positive association between high SATB2-Ir in colon cancer and cancer-specific survival and overall survival, whereas no prognostic value was seen for SATB2 expression in rectal cancer [23]. Thus, the prognostic potential of SATB2 expression still needs further clarification.

## 4. Materials and Methods

### 4.1. Patients and Tissue Sample Collection

Tissue specimens were obtained from 104 patients with confirmed CRC (46 women and 58 men; mean age ± standard deviation, 67.9 ± 10.8 years old; range: 33–91 years; women: 69.59 ± 10.75, 44–90 years; men: 66.64 ± 10.63, 33–91 years) at the Hospital of the Ministry of Internal Affairs and Administration in Olsztyn (Poland) from 2010 to 2015. None of the recruited patients had previously undergone chemo-, radiotherapy, and suffered from a second neoplastic disease. An additional inclusion criterion was the completeness of data on the clinicopathological parameters considered. Clinical and demographic characteristics were gathered at the time of enrollment.

Samples of CRC and matched non-involved colon mucosa (from a resected, macroscopically unchanged portion, at least 5 cm away from the tumor) were collected from patients during colectomy. Specimens for histological and IHC evaluation (*n* = 104 patients) were fixed in 10% neutral buffered formalin solution and further processed into paraffin blocks. Tissue samples for RNA extraction and qPCR analysis (*n* = 66 patients) were snap-frozen in liquid nitrogen and stored at −80 °C.

### 4.2. Total RNA Isolation, Reverse Transcription, and QPCR

Total RNA was isolated from the tissue samples and reverse transcribed as previously described by Kowalczyk et al. [53]. *SATB2* mRNA levels were determined by qPCR using the ABI 7500/7500 Fast Real-Time PCR System (Life Technologies, Applied Biosystems, Foster City, CA, USA), and normalized to that of β-actin (*ACTB*) and hypoxanthine phosphoribosyltransferase 1 (*HPRT1*) mRNA content. The amplification primer pairs used in qPCR are listed in Table 4.

Reactions were performed according to the previously described protocol [54]. The following thermocycling conditions were applied: initial denaturation at 95 °C for 20 s, followed by 40 cycles of denaturation at 95 °C for 3 s, and annealing and elongation at 58 °C for 40 s (*SATB2*), 62 °C for 40 s (*HPRT1*), or 60 °C for 40 s (*ACTB*). The ΔΔCt method [55] was used to estimate the fold differences (relative quantification (RQ)) in *SATB2* expression between the matched samples of CRC and non-involved colorectal tissues. Fold increase above 1 (RQ > 1) indicated *SATB2* overexpression in CRC tissue, and fold decrease under 1 (RQ < 1) indicated *SATB2* downregulation.

### 4.3. Immunohistochemistry and Staining Evaluation

Immunostaining of SATB2 in CRC and non-involved colon tissues was carried out as previously described [54] using 4 μm-thick paraffin sections and rabbit monoclonal primary antibodies against SATB2 (1:100, EPNCIR130A, ab92446, Abcam, Cambridge, UK). Negative controls were run in parallel, omitting the primary antibody. Immunostaining was accomplished using Autostainer Link 48 (DakoCytomation, Glostrup, Denmark).

SATB2-Ir was assessed using an Olympus BX53 light microscope (Olympus, Tokyo, Japan) by two independent pathologists blinded to the patients’ clinicopathological data. Only nuclear SATB2 staining was regarded as specific. The scoring system was based on the percentage of SATB2-positive enterocytes of normal colonic mucosa or SATB2-positive CRC cells (0, absence of staining; 1, when 1–10% cells were immunoreactive; 2, 11–50%; 3, 51–80% and 4, >80%). Based on the median SATB2-Ir value, CRC samples with an immunoexpression score ≤ 2 were considered to have “low” expression, while those with a score > 2 were considered to have “high” SATB2 expression.

### 4.4. Statistical Analysis

Statistical analyses were performed using STATISTICA 10 (version 10.0; StatSoft, Tulsa, OK, USA) and Prism 6 (version 6.07; GraphPad, La Jolla, CA, USA) software. Differences in *SATB2* mRNA and protein levels between the matched CRC and non-involved tissue samples were examined using a paired t test. Associations between *SATB2* gene expression levels and demographic and clinicopathological parameters were assessed by the Fisher’s exact test and confirmed using the unpaired t test and one-way ANOVA followed by Tukey’s multiple comparison test. Survival curves were plotted in accordance with the Kaplan–Meier method. The significance of differences in survival between patient groups based on different variables was assessed using the log-rank test and the Cox regression method. *p*-values < 0.05 were considered statistically significant.

## 5. Conclusions

The reduced expression of the *SATB2* gene in CRC tissues compared to paired normal, uninvolved large intestine tissues, as well as its decreased expression in high-grading tumors, support a tumor suppressor role of SATB2 and involvement of its impaired expression in the pathogenesis of CRC. The associations of SATB2 expression with patient gender and tumor location demonstrated in the present study, as well as the numerous discrepancies in the results of previous studies, indicate the complexity of the regulation of SATB2 expression in the colon and CRC tissues. Therefore, the significance of altered *SATB2* expression in CRC and the mechanisms of its regulation should be investigated in further studies.

## Figures and Tables

**Table 1:** Relationships between the relative *SATB2* mRNA expression and demographic and clinicopathological parameters of CRC patients.

		*SATB2* mRNA RQ (CRC vs. Non-Involved Colon Tissue)		
Parameters	Number of Cases	RQ < 1 *n* (%)	RQ > 1 *n* (%)	*p*-Value ^a^
Total	66	46 (69.7)	20 (30.3)	
Sex				
Men	34	24 (70.6)	10 (29.4)	1.0000
Women	32	22 (68.7)	10 (31.3)	
Localization				
Cecum, ascending and transverse colon	24	20 (83.3)	4 (16.7)	0.1892
Descending and sigmoid colon	18	11 (61.1)	7 (38.9)	
Rectum	24	15 (62.5)	9 (37.5)	
Grade of differentiation (G)				
G2	60	41 (68.3)	19 (31.7)	0.6588
G3	6	5 (83.3)	1 (16.7)	
Depth of invasion (T status)				
T1 + T2	9	8 (88.9)	1 (11.1)	0.2573
T3 + T4	57	38 (66.7)	19 (33.3)	
Lymph node metastasis (N status)				
N0	35	27 (77.1)	8 (22.9)	0.1292
N1 + N2	31	19 (61.3)	12 (38.7)	
Distant metastasis (M status)				
M0	56	42 (75.0)	14 (25.0)	0.0555
M1	10	4 (40.0)	6 (60.0)	
TNM stage				
I + II	31	25 (80.6)	6 (19.4)	0.1068
III + IV	35	21 (60.0)	14 (40.0)	
Quantitative parameter		r		*p*-Value ^b^
Age ^c^	66	−0.0699		0.5769

CRC—colorectal cancer; RQ—relative quantification; ^a^ Fisher’s exact test; ^b^ Spearman correlation; ^c^ age of patients at diagnosis, range: 33–91 years.

**Table 2:** Relationships between SATB2 nuclear immunoreactivity in tumor cells and demographic and clinicopathological parameters of patients with colorectal cancer (CRC).

		Nuclear SATB2 Immunoreactivity in CRC Cells		
Parameters	Number of Patients	Score ≤ 2 *n* (%)	Score > 2 *n* (%)	*p*-Value ^a^
Total	104	30 (28.8)	74 (71.2)	
Sex				
Men	58	9 (15.5)	49 (84.5)	**0.0010**
Women	46	21 (45.7)	25 (54.3)	
Localization				
Cecum, ascending and transverse colon	39	17 (43.6)	22 (56.4)	**0.0365**
Descending and sigmoid colon	26	5 (19.2)	21 (80.8)	
Rectum	39	8 (20.5)	31 (79.5)	
Grade of differentiation (G)				
G2	96	24 (25.0)	72 (75.0)	**0.0068**
G3	8	6 (75.0)	2 (25.0)	
Depth of invasion (T status)				
T1 + T2	16	6 (37.5)	10 (62.5)	0.5489
T3 + T4	88	24 (27.3)	64 (72.7)	
Lymph node metastasis (N status)				
N0	56	14 (25.0)	42 (75.0)	0.3904
N1 + N2	48	16 (33.3)	32 (66.7)	
Distant metastasis (M status)				
M0	91	28 (30.8)	63 (69.2)	0.3384
M1	13	2 (15.4)	11 (84.6)	
TNM stage				
I + II	52	13 (25.0)	39 (75.0)	0.5165
III + IV	52	17 (32.7)	35 (67.3)	
Quantitative parameter		r		*p*-Value ^b^
Age ^c^	104	−0.0076		0.9389

CRC—colorectal cancer; ^a^ Fisher’s exact test; ^b^ Spearman correlation; ^c^ age of patients at diagnosis, range: 33–91 years. Significant *p*-values (<0.05) are in bold.

**Table 3:** Univariate and multivariate Cox regression analyses of the overall survival rates related to different prognostic variables in patients with colorectal cancer (CRC).

Parameter	Univariate Analysis			Multivariate Analysis		
	HR	95% CI	*p*-Value	HR	95% CI	*p*-Value
*SATB2* mRNA RQ	0.98	0.88–1.08	0.6406			
SATB2 immunoreactivity in CRC	1.02	0.85–1.22	0.8492			
Sex (women vs. men)	0.80	0.44–1.46	0.4648			
Age (years) ^a^	1.04	1.01–1.07	**0.0082**	1.04	1.01–1.08	**0.0140**
Localization						
(rectum vs. cecum, ascending and transverse colon)	1.42	0.71–2.85	0.4221			
(descending and sigmoid colon vs. cecum, ascending and transverse colon)	1.56	0.72–3.38	0.6825			
Grade of differentiation (G3 vs. G2)	1.02	0.31–3.28	0.9776			
Depth of invasion (T3 + T4 vs. T1 + T2)	4.77	1.16–19.73	**0.0308**	1.87	0.42–8.28	0.4098
Lymph node metastasis (N1 + N2 vs. N0)	3.56	1.88–6.74	**0.0001**	2.30	0.54–9.69	0.2582
Distant metastases (M1 vs. M0)	7.09	3.48–14.4	**<0.0001**	4.84	1.98–11.78	**0.0005**
TNM stage (III + IV vs. I + II)	5.34	2.62–10.86	**<0.0001**	1.58	0.31–7.97	0.5784

HR—hazard ratio; RQ—relative quantification; CI—confidence interval. Significant *p*-values (*p* < 0.05) are in bold. ^a^ Age of patients at diagnosis, range: 33–91 years.

**Table 4:** Primer sequences used in quantitative real-time polymerase chain reaction.

Gene	Primer Sequences
*SATB2*	F: 5′-AGGAGTTTGGGAGATGGTAT-3′
	R: 5′-CCCAGAACACAATAGTCTGAA-3′
*ACTB*	F: 5′-TGTGCCCATCTACGAGGGGTATGC-3′
	R: 5′-GGTACATGGTGGTGCCGCCAGACA-3′
*HPRT1*	F: 5′-GACTTTGCTTTCCTTGGTCAGGC-3′
	R: 5′-TGGCGATGTCAATAGGACTCCAG-3′

F—forward primer, R—reverse primer.

## Data Availability

The data presented in this study are available from the corresponding author upon reasonable request.

## Abbreviations

The following abbreviations are used in this manuscript:

SATB2	Special AT-rich sequence-binding protein 2
CRC	Colorectal cancer
qPCR	Quantitative polymerase chain reaction
IHC	Immunohistochemical
HCC	Hepatocellular carcinoma
HNSCC	Head and neck squamous cell carcinoma
EMT	Epithelial-mesenchymal transition
OS	Overall survival
SATB2-Ir	SATB2-immunoreactivity
RQ	Relative quantification
HR	Hazard ratio
CI	Confidence interval
MANECs/NECs	Mixed adenoneuroendocrine carcinomas/neuroendocrine carcinomas
*lncRNA SATB2-AS1*	Long non-coding RNA antisense transcript of *SATB2*
TMA	Tissue microarray
miRs	MicroRNAs
MSI	Microsatellite instability
KRAS	Kirsten rat sarcoma virus
ACTB	β-actin
HPRT1	Hypoxanthine phosphoribosyltransferase 1
